# Study of montelukast in children with sickle cell disease (SMILES): a study protocol for a randomised controlled trial

**DOI:** 10.1186/s13063-021-05626-6

**Published:** 2021-10-10

**Authors:** Anna M. Hood, Hanne Stotesbury, Melanie Kölbel, Michelle DeHaan, Michelle Downes, Jamie M. Kawadler, Satwinder Sahota, Dagmara Dimitriou, Baba Inusa, Olu Wilkey, Maria Pelidis, Sara Trompeter, Andrea Leigh, Janine Younis, Emma Drasar, Subarna Chakravorty, David C. Rees, Sue Height, Sarah Lawson, Johanna Gavlak, Atul Gupta, Deborah Ridout, Christopher A. Clark, Fenella J. Kirkham

**Affiliations:** 1 Developmental Neurosciences Unit and Biomedical Research Centre, UCL Great Ormond Street Institute of Child Health, 30 Guilford Street, London, WC1N 1EH UK; 2 School of Psychology, University College Dublin, Dublin, Ireland; 3 Department of Psychology and Human Development, UCL Institute of Education, London, UK; 4 Children’s Sickle Cell and Thalassaemia Centre, Evelina London Children’s Hospital, Guy’s and St Thomas’ NHS Trust, London, UK; 5 North Middlesex Hospital National Health Service Trust, London, UK; 6 Department of Paediatric Haematology, Evelina London Children’s Hospital, Guy’s and St Thomas’ NHS Trust, London, UK; 7 University College London Hospitals NHS Foundation Trust, London, UK; 8 NHS Blood and Transplant, London, UK; 9 Whittington Health NHS Trust, London, UK; 10 Department of Clinical Haematology, University College London Hospitals NHS Foundation Trust, London, UK; 11 Paediatric Haematology, King’s College Hospital NHS Trust, London, UK; 12 Department of Haematological Medicine, King’s College Hospital NHS Trust, London, UK; 13 Birmingham Women’s and Children’s NHS Foundation Trust, Birmingham, UK; 14 Department of Child Health, University Hospital Southampton NHS Foundation Trust, Southampton, UK; 15 Department of Paediatric Respiratory Medicine, King’s College Hospital NHS Foundation Trust, London, UK; 16 Population, Policy and Practice Programme, UCL Great Ormond Street Institute of Child Health, London, UK; 17 Clinical and Experimental Sciences, University of Southampton, Southampton, UK

**Keywords:** Sickle cell anaemia, Processing speed, Executive function, Sleep-disordered breathing, hypoxia, Randomised control trial

## Abstract

**Background:**

Young children with sickle cell anaemia (SCA) often have slowed processing speed associated with reduced brain white matter integrity, low oxygen saturation, and sleep-disordered breathing (SDB), related in part to enlarged adenoids and tonsils. Common treatments for SDB include adenotonsillectomy and nocturnal continuous positive airway pressure (CPAP), but adenotonsillectomy is an invasive surgical procedure, and CPAP is rarely well-tolerated. Further, there is no current consensus on the ability of these treatments to improve cognitive function. Several double-blind, randomised controlled trials (RCTs) have demonstrated the efficacy of montelukast, a safe, well-tolerated anti-inflammatory agent, as a treatment for airway obstruction and reducing adenoid size for children who do not have SCA. However, we do not yet know whether montelukast reduces adenoid size and improves cognition function in young children with SCA.

**Methods:**

The Study of Montelukast In Children with Sickle Cell Disease (SMILES) is a 12-week multicentre, double-blind, RCT. SMILES aims to recruit 200 paediatric patients with SCA and SDB aged 3–7.99 years to assess the extent to which montelukast can improve cognitive function (i.e. processing speed) and sleep and reduce adenoidal size and white matter damage compared to placebo. Patients will be randomised to either montelukast or placebo for 12 weeks. The primary objective of the SMILES trial is to assess the effect of montelukast on processing speed in young children with SCA. At baseline and post-treatment, we will administer a cognitive evaluation; caregivers will complete questionnaires (e.g. sleep, pain) and measures of demographics. Laboratory values will be obtained from medical records collected as part of standard care. If a family agrees, patients will undergo brain MRIs for adenoid size and other structural and haemodynamic quantitative measures at baseline and post-treatment, and we will obtain overnight oximetry.

**Discussion:**

Findings from this study will increase our understanding of whether montelukast is an effective treatment for young children with SCA. Using cognitive testing and MRI, the SMILES trial hopes to gain critical knowledge to help develop targeted interventions to improve the outcomes of young children with SCA.

**Trial registration:**

ClinicalTrials.govNCT04351698. Registered on April 17, 2020. European Clinical Trials Database (EudraCT No. 2017-004539-36). Registered on May 19, 2020

## Administrative information

Note: the numbers in curly brackets in this protocol refer to SPIRIT checklist item numbers. The order of the items has been modified to group similar items (see http://www.equator-network.org/reporting-guidelines/spirit-2013-statement-defining-standard-protocol-items-for-clinical-trials/).

**Table:** 

Title {1}	Study of Montelukast in Children with Sickle Cell Disease (SMILES): protocol for a randomised controlled trial
Trial registration {2a and 2b}.	SMILES: Study of Montelukast in Sickle Cell Disease (SMILES). ClinicalTrials.gov, NCT04351698, registered on April 17, 2020; European Clinical Trials Database (EudraCT No. 2017-004539-36), registered on May 19, 2020
Protocol version {3}	05-16-2020; protocol version 1.6
Funding {4}	Action Medical Research (GN2509)
Author details {5a}	Developmental Neurosciences Unit and Biomedical Research Centre, UCL Great Ormond Street Institute of Child Health, London, United Kingdom
Name and contact information for the trial sponsor {5b}	Great Ormond Street Hospital for Children NHS Foundation Trust Joint R&D Office GOSH/ICH Great Ormond Street Hospital for Children NHS Foundation Trust UCL Great Ormond Street Institute of Child Health 30 Guilford Street London WC1N 1EH United Kingdom
Role of sponsor {5c}	The study sponsor (UCL Great Ormond Street Institute of Child Health) will manage funding. The trial sponsor supplies the infrastructure and enables the running of the trial but has no influence over study design; collection, management, analysis, and interpretation of data; writing of the report; and submitting the report for publication.

## Introduction

### Background and rationale {6a}

Sickle cell disease (SCD) is the collective term for a group of autosomal recessive haemoglobinopathies in which haemoglobin polymerises after de-oxygenation, causing red blood cells (RBCs) to become sticky, rigid, and sickle-shaped [1]. These properties account for the clinical manifestations of SCD, which include anaemia and jaundice [2]. Approximately 3.5% of the world population carries the pathological haemoglobinopathy gene; however, SCD is most common in individuals of African ancestry [3, 4]. Medical advances have altered the natural course of SCD from acute childhood illness with high mortality to a chronic disease with associated multi-organ complications, significant disability, morbidity, and enormous health and economic burden [5]. As life expectancy has improved because of substantial advances in prophylactic and potentially curative treatment options [4, 6], there is more emphasis on amelioration of complications, including overt stroke [7], silent cerebral infarct (SCI) [8, 9], and cognitive difficulties [10, 11], for which there is evidence for an association with lower oxygen saturation (SO_2_) [12].

The frontal lobes have a protracted period of development in comparison to other brain regions and are often the site of overt stroke and SCI, which can affect young children with SCD [8, 9]. Relatedly, cognitive impairments appear early, are often detectable in the first 3 years [13–17], and may become worse with age [18, 19], even in those with no clinical history or magnetic resonance imaging (MRI) indication of stroke or SCI [10]. Although cognition encompasses various domains, children with SCD appear to experience particular difficulties with executive function [20, 21], processing speed [22], and attention [23, 24]. Evidence that airway obstruction and continuous exposure to low SO_2_ may increase the risk of stroke and SCI [25–29], poorer brain white matter integrity [28], and cognitive difficulties [12, 30, 31], has been accumulating. SO_2_ often falls during sleep and results in sleep-disordered breathing (SDB), especially if airway obstruction is related to the size of the adenoids in the nose, the tonsils, and in the throat.

SDB is characterised by abnormal respiratory patterns or pauses in breathing and insufficient ventilation during sleep, and there is evidence that inflammation plays a role [32]. Obstructive SDB in young children is typically caused by adenotonsillar hypertrophy [33]. Common treatments for SDB include timely adenotonsillectomy [34, 35] and long-term nocturnal continuous positive airways pressure (CPAP) [31, 36]. Both treatments have shown some effectiveness in reducing symptoms of SDB; however, adenotonsillectomy is an invasive procedure that requires anaesthesia and has potentially life-threatening complications. In addition, many children continue to suffer from persistent SDB after surgery [37]. Following CPAP treatment, improvements in cognition have been demonstrated in children with SDB, but it remains uncertain whether these improvements are sustained [35, 38–40]. There are limited data from randomised trials of treatment for SDB with cognitive endpoints in those with SCD even though SDB prevalence is higher [41], and the risk of hypoxic exposure is greater [42, 43]. Data from a pilot phase 1 randomised controlled trial (RCT) found that 6 weeks of auto-adjusting CPAP improved on the Wechsler Cancellation subtest, a measure of processing speed and attention, in school-age children with SCD [31, 44]. Preliminary data from the phase-2 trial also show a trend for improved processing speed in children with SCD aged over 8 years randomised to auto-adjusting CPAP [45, 46]. These findings suggest that interventions targeting hypoxic exposure may improve cognitive difficulties for children with SCD. However, although CPAP is non-invasive, adherence and acceptability problems such as patient discomfort, nasal congestion, and perceived difficulty with portability limit long-term usefulness [47].

Previous research has demonstrated that the leukotriene pathway appears to be involved in adenotonsillar inflammation [48, 49]. Therefore, montelukast, a leukotriene antagonist and a non-invasive, well-tolerated orally administered drug, represents a promising alternative treatment to CPAP for children with SDB. The efficacy and anti-inflammatory properties of montelukast have been shown in the treatment of several paediatric respiratory conditions, including asthma [50], exercise-induced bronchospasm [51, 52], and allergic rhinitis [37, 53, 54]. Double-blind RCTs have also demonstrated the efficacy of montelukast compared to placebo in non-SCD paediatric patients with SDB and asthma [55–58], even in children as young as 2 years [59], but these studies have not included cognitive outcomes. There may be critical periods in childhood where early intervention might prevent or reverse cognitive function deterioration [60]. As such, montelukast is a potential early intervention that could ameliorate SDB, along with the associated vulnerability in brain white matter and impairments in processing speed and executive function observed in young children with SCD.

### Objectives {7}

The Study of Montelukast In Children with Sickle Cell Disease (SMILES) trial plans to gain critical knowledge to develop targeted interventions to improve the outcomes of young children with sickle cell anaemia (SCA). For this trial, SCA will include children with both the HbSS and HbSβ_0_ thalassaemia genotypes [61]. Using cognitive testing and MRI, we will investigate plausible, modifiable endpoints [62–65] at an age (3.00–7.99 years) when compromise of brain structure and cognitive function is potentially preventable or reversible [60]. The primary objective of the SMILES trial is to assess the effect of montelukast on the processing speed of young children with SCA. Secondary objectives include evaluating the impact of montelukast on executive function, adenoidal size, brain volumetrics, white matter integrity, structural and functional connectivity, perfusion and oxygenation (measurable on MRI) on sleep, respiratory, and pain symptoms. Additionally, we will monitor any side effects of montelukast and the feasibility of non-sedated MRI in young children with SCA.

#### Primary hypothesis

Young children with SCA + SDB receiving montelukast will have improved processing speed compared to those receiving placebo.

#### Secondary hypothesis (i)

Young children with SCA + SDB receiving montelukast will have improved executive function compared to those receiving placebo.

#### Secondary hypothesis (ii)

Young children with SCA + SDB receiving montelukast compared with those receiving placebo will have improved overnight oximetry measures, fewer asthma symptoms, and reduced adenoidal size on MRI.

#### Secondary hypothesis (iii)

Young children with SCA + SDB have abnormal brain imaging (e.g. SCI and/or reduced white matter integrity) related to the severity of SDB, which will improve in those treated with montelukast compared with those receiving placebo.

### Trial design {8}

**Fig. 1:**
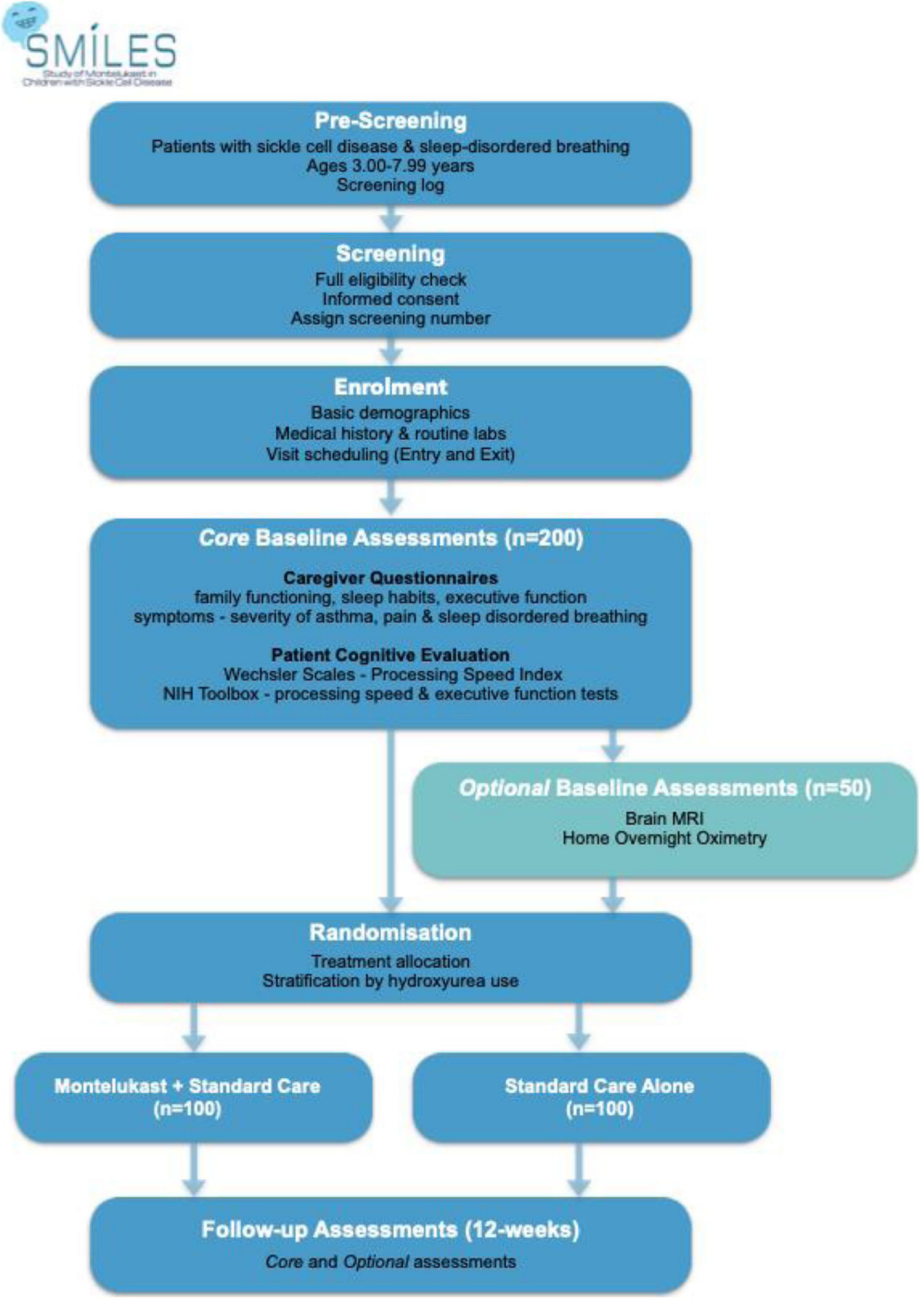
The Study of Montelukast in Children with Sickle Cell Disease (SMILES) Trial Flow Chart

SMILES is a parallel group, 12-week prospective, multicentre, double-blind, randomised controlled trial. This superiority trial investigates the extent to which montelukast can improve processing speed, reduce adenoidal size and white matter damage, and therefore, improve symptoms or signs of SDB compared to placebo for young children (3–7.99 years) with SCA. Children younger than 3 years will not be tested so that patients can complete the same age-appropriate cognitive tests. Patients will be randomised to receive either 4 mg of oral, chewable montelukast once daily at night (Treatment Arm) or oral placebo (Control Arm) with one-to-one allocation. Families will meet with the research team before the evaluation at Great Ormond Street Hospital for Children (GOSH), at clinics at participating sites, or school/home before randomisation to complete cognitive testing and caregiver proxy reports of child sleep and pain symptoms (see Fig. 1).

## Methods: participants, interventions, and outcomes

### Study setting {9}

Patients will be recruited from participating sites in the UK (i.e. North Middlesex Hospital, Evelina Children's Hospital, Whittington Health, University College London Hospital, and King's College Hospital). The research team will undertake trial procedures at GOSH.

### Eligibility criteria {10}

Eligibility screening will involve a checklist of items relating to inclusion and exclusion criteria (see Table 1). A research coordinator will administer the checklist once they have obtained written consent. The Chief Investigator (CI) or qualified doctor delegated for consent in Clinical Trial of an Investigational Medicinal Product (CTIMPs) will confirm eligibility. An identification number will be issued and used on all subsequent record forms. If the participant withdraws or is not eligible after screening, the identification number will not be re-used. Relevant clinical history will be taken. A potential participant can proceed to entry if they, their caregivers/guardian, and their treating clinician have agreed to randomisation and if all other eligibility criteria are fulfilled and checked by the CI. If a patient has significant SDB symptoms based on history and observation of obstructive pattern, they can be included in the trial if they are waiting for adenotonsillectomy (the current waiting list for non-urgent surgeries is 18 weeks) or if they do not need surgery. Children receiving chronic blood transfusion (> 3 months) or hydroxyurea treatment are also eligible for the study.

**Table 1:** Participant eligibility criteria for participation in the SMILES trial

Inclusion criteria	Exclusion criteria
Aged between 3.00 and 7.99 years	Developmental or psychiatric disorders including craniofacial abnormalities, neuromuscular disorder, and other chronic conditions
HbSS or HbSβ_0_ thalassaemia diagnosed by standard techniques (HPLC, IEF, MS or alkaline electrophoresis, DNA analysis, family studies)	Patient already taking/prescribed montelukast
History of sleep-disordered breathing (SDB), (i.e. caregiver-reported symptoms of SDB or any abnormality on overnight oximetry compared with published data in children of the same age (e.g. nadir SaO_2_ < 93%; mean SaO_2_ < 96%))	The patient has experienced side effects or an adverse reaction to montelukast at any time the past
Patient in a steady-state (i.e. haemoglobin not decreased by > 10% during the previous year, no painful episodes requiring opioids for at least 1 month and at least 1 month after any hospital admission)	Patient on CYP 3A4, 2C8, and 2C9 inducers, such as phenytoin, phenobarbital, and rifampicin
Likely to comply with treatment for 3 months as determined by the local paediatrician or haematologist	Presence or history of another haemoglobinopathy or blood dyscrasia
Able to speak and understand English	Concomitant treatment with any other leukotriene antagonist within the enrolment period
	Enrolled concurrently in any other SCA/montelukast trials

### Who will take informed consent? {26a}

The local site Principal Investigator (PI) or other appropriately medically trained site staff (delegated by the PI) at the site are responsible for assessing the caregiver/legal guardians’ ability to provide written informed consent. Members of the research team will ensure that the caregiver/legal guardian is fully informed about the trial and will discuss the current approved patient information sheet. The CI, local PI, or appropriately medically trained site staff (have received GCP training and certified) will obtain written consent/assent at a mutually convenient time at GOSH or the local recruitment site after explaining the aims, methods, and benefits and potential risks of the trial in detail. They will obtain written informed consent on the currently approved version of the trial consent form before conducting any trial-specific procedures.

### Additional consent provisions for collection and use of participant data and biological specimens {26b}

If new safety information results in significant changes in the risk/benefit assessment, the consent form will be reviewed and updated if necessary, and participants will be re-consented as appropriate. This trial does not involve collecting biological specimens for storage.

### Interventions

#### Explanation for the choice of comparators {6b}

As there are no evidence-based guidelines for managing OSA in children with SCA, the comparator will be matched placebo stratified by whether the patient is receiving hydroxyurea.

#### Intervention description {11a}

Montelukast is a leukotriene receptor antagonist that binds to the cysteinyl leukotriene receptor type 1 in the lung bronchial tubes and adenotonsillar tissues, blocking the action of leukotriene D4 [66]. This reduces the bronchial-constriction that leukotriene activity otherwise leads to, reducing inflammation. Montelukast is rapidly absorbed following oral administration. In the fasted state, mean peak plasma concentration (Cmax) is achieved 2 h after administering the 4 mg chewable tablet for patients aged 2 to 5 years. We based regimen and dose selection on current evidence-based National Institute for Clinical Excellence (NICE) guidelines for montelukast asthma use [67]. The results of recent trials of montelukast demonstrated improvement in obstructed sleep apnoea (OSA) for young children in the general population [57, 68, 69].

Although montelukast has a marketing authorisation, we will use it outside the manufacturer’s indication in this study. Therefore, this trial’s impact project management (IMP) risk is categorised as Type B ‘somewhat higher than that of standard medical care’. Guys and St. Thomas Hospital (GSTH) will manufacture montelukast and the matched placebo under their MIA (IMP) license 11387 and will be released by a qualified person at GSTH. The investigational medicinal product will be manufactured in three batches as per requirement. The GOSH pharmacy must notify the trial coordinator at least 2 months before if they need additional supply. Each bottle of montelukast and matched placebo contains 31 tablets. Each bottle will have a unique pack ID number (Kit number) and a portion label that the pharmacist will need to tear off at the time of dispensing to each patient. There will be only one dispensing of 3 bottles per patient. The CI will decide whether to replace lost or damaged supply.

#### Criteria for discontinuing or modifying allocated interventions {11b}

The CI and local PI in consultation with the respiratory physician (AG; author) may withdraw participants from the trial if they consider that the risk of continuing in the trial is too great, for example, if the child is experiencing side effects associated with montelukast or if they require montelukast to treat asthma symptoms. Each caregiver or legal guardian can choose to withdraw a participant from the trial at any time. If a caregiver or legal guardian expresses their wish to withdraw from the trial, the site research staff will first explain the importance of remaining in the trial. If they still wish to withdraw, the research staff will request that the collected data still be used. If a patient or guardian explicitly states they do not want to contribute further data to the trial, their decision will be respected. Research staff will record withdrawals and the patient’s reason on a case report form, and the local PI will note in the patient’s hospital records. If a participant chooses to discontinue their trial treatment, they will continue to be followed as closely as possible to the follow-up schedule defined in the protocol, providing they are willing. If the participant is withdrawn due to an adverse event, the CI will arrange for follow-up visits or telephone calls until the adverse event has resolved or stabilised. We will not replace participants who stop trial follow-up early. We do not anticipate any dose adjustments during the trial.

#### Strategies to improve adherence to interventions {11c}

We will request that caregivers return all three bottles of montelukast or the placebo (even if empty or unused) to the GOSH pharmacy for accountability. Caregivers will complete a medical adherence questionnaire (see secondary outcomes) with the assistance of the clinical research coordinator during the 2- and 6-week phone calls (see Table 2).

**Table 2:** Details of study flow

Procedures	Recruitment VA/B	Baseline V1 (week 0)	Phone call (week 2)	Phone call (week 6)	Follow-up V2 (week 12)	Phone call (week 14)
Informed consent	X					
Recent labs	X					
Medical history	X					
Eligibility assessment	X					
Demographics		X				
Family functioning questionnaires		X				
Concomitant medications		X			X	
Questionnaires		X			X	
Cognitive testing		X			X	
MRI if consenting and feasible		(X)			(X)	
Home oximetry if consenting and feasible		(X)			(X)	
Randomisation		X				
Dispensing of trial drugs		X				
Drug accountability					X	
Adverse event assessments			X	X	X	X

#### Relevant concomitant care permitted or prohibited during the trial {11d}

Most children with SCA are prescribed folic acid and penicillin. Hydroxyurea is a disease-modifying treatment for patients with SCA, which has many beneficial effects, including reduced pain and acute chest syndrome episodes, reduced hospital admissions, and less need for blood transfusions [70, 71]. Children enrolled in the trial prescribed folic acid, penicillin, and hydroxyurea will continue this treatment. The research coordinator will track any changes in hydroxyurea status during the trial (e.g. discontinuation). The treating physician can make any changes they deem clinically necessary, and we will account for these differences in statistical analyses. However, as the trial duration is 3 months, we anticipate that any changes will affect only a small number of patients. There is no evidence of interaction between montelukast and any of these three drugs [72].

#### Provisions for post-trial care {30}

There will not be a provision of the IMP beyond the trial period unless the local paediatrician haematologist or respiratory physician considers (at the discretion of the treating physician) that montelukast should be prescribed on clinical grounds (e.g. the child is diagnosed with asthma). If prescribed, it will be done according to current standard care guidelines rather than the trial supply.

#### Outcomes {12}

The research team will collect demographic, education, and health information from medical records, and participants’ caregivers will complete questionnaires. Caregivers will complete three questionnaires related to sleep as they capture related but distinct information about sleep habits, sleep-disordered breathing, and general sleepiness. We will obtain cognitive data (processing speed, executive function) from performance on tasks administered via a tablet computer. We will calculate both raw and norm-calculated standard scores (Mean = 100; SD = 15) in cognitive data analyses. We will calculate the Processing Speed Index (PSI) from the Wechsler subtests administered using scores from two subtests (substituting the third test if necessary). Executive function scores will be calculated from the NIH Toolbox® for Assessment of Neurological and Behavioral Function (NIH Toolbox®) Cognition Module [73] to obtain a composite that has been used in previous research [20]. We will obtain laboratory values (e.g. complete blood count, haemoglobin analysis, HbS%, HbF%) collected as standard care from medical records. When possible, we will collect data regarding brain function using MRI techniques and data regarding sleep from home oximetry (see Table 2 for the trial assessment schedule).

The primary and secondary outcomes have clinical relevance as they represent many of the significant co-morbidities (e.g. slowed processing speed, reduced white matter integrity, respiratory symptoms, sleep disturbances, pain, and low quality of life) experienced by paediatric patients with SCA [22, 45, 74–76]. The cognitive tests and questionnaires have been validated in numerous previous studies, often with a paediatric SCA population. The primary and secondary outcomes’ potential risks are minimal and only include those associated with routine cognitive testing and MRI scans. Testing procedures may induce boredom, fatigue, emotional discomfort, anxiety, or frustration. There is a rare risk that we may identify a previously undiagnosed learning disorder during the study. If that is the case, we will notify the caregiver and refer them to a proper specialist. We will screen participants for contraindications to MRI. If contraindications are found, the participant will not undergo MRI but can complete other study procedures if willing.

Participant screening will occur on each day that they come for an MRI session. We will attempt to minimise any anxiety related to MRI by thoroughly explaining the procedures and the MRI scanner to participants before the study. Participants will view a video explaining the nature of the study and participate in a mock scanning session. During the scanning procedures, the participants are continually monitored visually and aurally for any potential problems. The research staff will assure participants that they can be removed from the scanner at any time if problems should arise or they are experiencing discomfort. The risks of PSG recording include minor skin irritations from the electrodes. Blood draws are components of standard clinical care and, therefore, do not add additional risk to participants.

#### Participant timeline {13}

See Table 2.

#### Sample size {14}

Our sample will include 200 patients with SCA (i.e. HbSS or HbSβ^0^ thalassaemia) with some evidence of sleep-disordered breathing (e.g. snoring and overnight oximetry outside the normal range) aged between 3 and 7.99 years (see Table 1 for a full description of eligibility criteria). The primary outcome for this study is processing speed. We chose this outcome based on our previous research in SDB alone [77] and current preliminary findings in children with SCA and sleep-disordered breathing [46, 78]. Scores on the tests of processing speed are normally distributed (Mean = 100; SD = 15). Our study of preschool children with SCA showed slightly greater variability (SD = 16), and so this value was used in the sample size calculations to give a more conservative estimate. Statistical analyses determined that two groups of 100, one on montelukast and one on placebo, will give 90% power to detect a standardised difference of 0.5 at the 2.5% significance level, which equates to 8 points assuming an SD of 16. This difference is of clinical significance.

#### Recruitment {15}

The CI and research team have conducted several successful multisite studies and previous clinical trials with caregivers and patients with SCA [45, 46, 79]. Because of these existing relationships with the study population, the research team has significant ties to the community, charitable organisations that support patients, and caregiver and patient stakeholders. Therefore, we anticipate that we can meet recruitment goals. If we cannot recruit 200 participants, with a minimum recruitment rate of at least 50%, we will have > 90% power to determine whether the adenoid to nasopharynx ratio reduces in children with SCA taking montelukast [80]. Previous studies have shown a large reduction in adenoid to nasopharynx ratio in 23 children with SCA taking montelukast (Mean = 0.81, SD = 0.04 pre-treatment vs Mean = 0.57, SD = 0.04 post-treatment) compared with no change in the placebo control group of 23 children [80].

### Assignment of interventions: allocation

#### Sequence generation {16a}

Each participant will either be receiving hydroxyurea or standard care, which will form a 2-category stratification variable. A computer programme (SIMIN package) will generate a stratified blocked random allocation sequence with randomly assigned block sizes (4, 6, 8, or) to ensure suitable allocation concealment. There will be an equal allocation distribution between montelukast (treatment) and placebo (control).

#### Concealment mechanism {16b}

Only the pharmacist undertaking the randomisation will know whether the patient has been allocated to montelukast or placebo. Patients are allocated to groups by minimization with weighting, which ensures that the groups are balanced with respect to prognostic indicators (minimisation criteria) that are likely to affect outcomes and is concealed from the investigators and families. The GOSH pharmacy will use the patient’s hospital number to record the allocation, and at the end of the trial, they will pass this information to the study statistician. At the end of the trial, the pharmacist will check that the current sequence is the same as what was initially generated, and they will note and query any discrepancies.

#### Implementation {16c}

A research team member will be responsible for setting up the randomisation system and passing the resulting sequence list to pharmacists at GOSH, who will use this to allocate patients as they present for the prescription. This team member will not enrol or assign participants to the intervention or conduct cognitive and MRI evaluations. Consecutive eligible children with SCA will be offered the study from selected sites via their consultant paediatrician or haematologist or ENT surgery waiting lists or their local sleep laboratory; non-enrollers will be logged. The pharmacist will dispense the intervention during the patient’s visit for cognitive evaluation and MRI. The pharmacist will otherwise have no patient contact.

### Assignment of interventions: blinding

#### Who will be blinded {17a}

The CI, local PIs, and researchers involved in data collection will be blind to group allocation. Pharmacists will be unblinded for dispensing purposes. Statisticians will be unblinded to allocation before preparation of the participant flow diagram and outcome analysis.

#### Procedure for unblinding if needed {17b}

The code breaks for the trial are held in GOSH pharmacy and are their responsibility. If the CI needs to know if a patient has been prescribed montelukast or placebo to manage a specific serious adverse event, the CI may be unblinded for that particular participant. The responsibility to break the treatment code in emergencies resides solely with the CI. The CI has unrestricted and immediate access to emergency envelopes available at the site to break the treatment code. Out of hours code-breaking will be possible if necessary. The GOSH pharmacy will put together a standard operating procedure (SOP) which will have information on unblinding out of hours. The on-call pharmacist will have access to a folder where the code will be available, and he/she will also have access to mobile numbers of the clinical trial team and the pharmacist. Data will be entered into a phone app that will link to an anonymised dataset locked in the UCL Data Safe Haven. Once this is complete, the statistician will receive the unblinded codes from the GOSH pharmacy for the statistical analysis.

### Data collection and management

#### Plans for assessment and collection of outcomes {18a}

At baseline, caregivers will complete measures of demographics (e.g. age, gender, ethnicity, parental education), pain burden, asthma symptoms, family functioning, child executive function, and sleep questionnaires. A clinical psychologist (AMH; first author) with significant assessment experience will train PhD candidates and paediatric neuropsychology MSc students to conduct the cognitive evaluation and assist caregivers with questionnaires. Assessors will administer cognitive testing to patients on a tablet computer. Given that proximity to a blood transfusion has been shown to influence cognition [20], we will test patients at the same time in their transfusion cycle at baseline and post-treatment (e.g. if we test a patient in the week before a transfusion at baseline, we will test a patient in the week before a transfusion at post-treatment).

Questionnaires and testing should take about 1 h to complete. If a family agrees (optional assessments), patients will undergo brain MRI after cognitive testing, and the research team will organise a reading of overnight oximetry. Patients will then be randomised to the control or treatment arm by the GOSH pharmacist. Montelukast will be immediately dispensed or posted to families. At the 12-week follow-up, caregivers and patients will complete questionnaires and cognitive testing again. Optional assessments will also be completed (see Fig. 1) Patients and families will complete an adverse event assessment and visit the GOSH pharmacy for montelukast accountability. We will compensate families for transportation (e.g. taxis) to and from the site for both visits. The study coordinator will call families at weeks 2, 6, and 14 to check for any adverse events (e.g. hospitalisations), focusing on potential patient behaviour changes (see Table 2) and medication adherence.

Given that this trial will be conducted in the UK during the coronavirus disease (COVID-19) pandemic, we aim to begin our cognitive evaluation in June 2021. We will conduct our cognitive evaluation in one of two ways. Families can choose from the two options. Families can choose to complete the cognitive evaluation in the same room as an assessor at a hospital site with guidelines to minimise COVID-19 infection (e.g. separate tables, masks, handwashing, enhanced cleaning of tablet computers). Families will also have the option to complete the cognitive evaluation in a different room to the assessor, with both facing a computer monitor with a camera. Caregivers will not be in the room but will be able to stand directly outside with another assessor. Caregivers can complete questionnaires during the cognitive evaluation, online (e.g. video conferencing), or by phone with a research team member’s assistance.

### Primary outcome

#### Processing speed

The Cancellation, Symbol Search, and Coding subtests from the Wechsler Preschool & Primary Scale of Intelligence Fourth Edition (WPPSI-IV) [81] and the Wechsler Intelligence Scale for Children Fifth Edition (WISC-V) [82] will be used to calculate our primary endpoint of processing speed. We will give all three processing speed Wechsler subtests to obtain a two subtest Processing Speed Index. We will use this testing strategy to ensure that we have two usable processing speed scores from all patients. We will choose the most developmentally appropriate test for patients. All tests will be administered using Q-interactive [83] on a tablet computer and following published guidelines for remote assessment (if testing in a separate room is necessary). Cancellation requires the child to scan a structured arrangement of coloured shapes and mark target shapes within a specified time limit. For the WPPSI-IV, processing speed subtests include Bug Search and Animal Coding. Bug Search requires the child to match visual stimuli. Animal Coding is a speeded paired-associates subtest in which the child marks shapes that correspond to animals. For the WISC-V, we will administer the analogous Symbol Search and Coding subtests.

For our second primary processing speed endpoint, we will administer the Pattern Comparison Test, a subtest in the Cognition Module of the NIH Toolbox®. The cognition module includes automated scoring; however, the assessor presents task instructions, monitors compliance, and ensures valid results. All instructions will be presented visually on the tablet screen using the iPad app with instructions spoken orally by the assessor. Participants use their index fingers to identify whether two side-by-side pictures shown on the tablet screen are the same (press the ‘Yes’ button) or are not (press the ‘No’ button) the same. Participants have 90 s to respond to as many pictures as possible. The number of correct responses completed will be scored, up to a maximum of 130.

### Secondary outcomes (patient)

#### Executive function

We will analyse two executive function tests from the Cognition Module of the NIH Toolbox® as secondary outcomes [73]. Scoring is a combination of accuracy and reaction time.

##### Dimensional Change Card Sort Test

Children will match targets to test pictures that vary along two dimensions (i.e. colour and shape). The test consists of four blocks (practice, pre-switch, post-switch, and mixed). Children will respond to targets as quickly as possible without making mistakes. There are 40 trials.

##### Flanker Inhibitory and Attention Test

Children will indicate the direction of a target and respond as quickly as possible without making mistakes. On congruent trials, flankers pointed in the same direction as the target. On incongruent trials, flankers pointed in the opposite direction as the target. For children aged 3–7 years, scores of ≥ 90% using preliminary fish stimuli are followed by an additional 20 trials using arrows.

### Secondary outcomes (caregivers)

#### *Behavioral Rating Inventory of Executive Function (BRIEF-2)* [84].

The BRIEF-2 is a caregiver-reported questionnaire with 63 items that assesses executive function behaviours in the school and home environments. The Global Executive Composite, Behavioral Regulation, and Emotion Regulation indices will be used in analyses. Higher *T*-scores (> 65) indicate more clinical concern.

#### Quality of Life

##### Pediatric Quality of Life Inventory Sickle Cell Disease Module (PedsQL) [85]

Caregivers will complete the PedsQL, a 43-item questionnaire with nine dimensions (pain and hurt, pain impact, pain management and control, worry, emotions, treatment, and communication). Caregivers rate how much of a problem an issue had been during the past month on a 5-point Likert scale of 0 = ‘Never’ to 4 = ‘Almost Always’. Items are reverse-scored and linearly transformed to a 0–100 scale (0 = 100, 1 = 75, 2 = 50, 3 = 25, 4 = 0), so that higher scores indicate better quality of life. Overall and subscale scores are computed as the sum of the items divided by the number of items answered. Clinical classifications for the PedsQL are as follows: 81–100 = low levels of pain, 61–80 = intermediate levels of pain, and 0–60 = poor HRQOL related to pain [86].

#### Sleep

##### Children’s Sleep Habit Questionnaire (CSHQ) [87]

Caregivers will complete the CSHQ, a 45-item caregiver-rated questionnaire: 33 scored questions and seven additional items intended to provide other relevant information about sleep behaviour (e.g. nocturnal body pains). The CSHQ assesses the frequency of behaviours associated with common paediatric sleep difficulties. Caregivers rate their child’s sleep habits and possible difficulties with sleep during the past week on a 3-point Likert scale: ‘usually’ (i.e. 5–7 times within the past week), ‘sometimes’ (i.e. 2–4 times within the past week), or ‘rarely’ (i.e. never or 1 time within the past week). A total sleep disturbances score is calculated as the sum of all CSHQ scored questions and can range from 33 to 99, with a score of over 41 indicating a paediatric sleep disorder.

##### Pediatric Sleep Questionnaire (PSQ) [88]

Caregivers will complete the PSQ, a 22-item questionnaire that asks about snoring frequency and loudness, observed apnoea, difficulty breathing during sleep, daytime sleepiness, and inattentive or hyperactive behaviour. Responses are ‘yes’ = 1, ‘no’ = 0, and ‘don't know’ = missing. The mean response to non-missing items is the score, which can vary from 0 to 1. The total ‘yes’ responses are summed, and a score of 8 or more indicates a further sleep evaluation may be necessary.

##### Epworth Sleepiness Scale (ESS) [89]

Caregivers will complete the ESS, an 8-item questionnaire that assesses the chance of dozing off or falling asleep whilst engaged in 8 different activities. Responses are rated on a 4-point scale of 0 = ‘would never doze’ to 3 = ‘high chance of dozing’. The ESS total score is the sum of all items and can range from 0 to 24. The higher the ESS score, the higher the child’s daytime sleepiness.

#### Asthma

##### Childhood Asthma Control Test (C-ACT) [90]

The C-ACT consists of 7 items of asthma control during the previous month. The C-ACT is divided into two sections. The child completes the first section, consisting of four questions on perception of asthma control, limitation of activities, coughing, and awakenings at night. Each question has four response options. The caregiver completes the second section, which consists of three questions (daytime complaints, daytime wheezing, and awakenings at night) with six response options. The sum of all scores yields the C-ACT score, ranging from 0 (poorest asthma control) to 27 (optimal asthma control). A cut-off point of more than 19 indicates uncontrolled asthma.

#### Adherence

##### Medical Adherence Questionnaire (MAQ) [91]

The MAQ is a 4-item measure where caregivers answer yes or no regarding items that assess their child’s adherence to montelukast during the SMILES trial. Items are summed to achieve a total adherence score.

### Optional secondary assessments

#### Brain MRI

We will conduct core MRI endpoints in a subset of children (*N* = 50) willing to undergo MRI. Patients and families will be offered a research MRI at GOSH. Upon arrival, the patient’s caregiver/guardian will complete an MRI safety checklist with an experienced radiographer. The MRI protocol will consist of conventional and advanced sequences to quantify any abnormality due to hypoxia/ischaemia. The protocol will be conducted in a research time slot, lasting under 60 min. Images will be checked for motion artefacts; data considered to be corrupted will be discarded. We use experienced paediatric radiographers to reduce the failure rate, which has been between 5 and 10% in previous studies. All MRI sequences will be without sedation and completely non-invasive. MRI protocol will be undertaken on a 3-T Siemens Prisma scanner.

##### Cerebrovascular disease

A T2-weighted fluid-attenuated inversion recovery (FLAIR) volume sequence (1 × 1 × 1 mm image resolution, 6 min duration) will be used to diagnose SCI.

##### Adenoidal size and brain volumetrics

A T1-weighted MPRAGE sequence (1 × 1 × 1 mm image resolution, 5-min duration) will be used to obtain high-resolution anatomical/volumetric information. Segmentation and parcellation through established processing pipelines using Freesurfer will provide measures of intracranial, white matter, grey matter volumes, and volumes of cortical and subcortical structures. The adenoidal to nasopharyngeal ratio will be measured from the sagittal (scout) view on T1 MRI.

##### Perfusion

A pulsed arterial spin-labelling sequence (3D single shot GRASE acquisition, matrix size = 64 × 64, 20 contiguous slices with 5 mm thickness, 6 min duration) will be used to assess perfusion. Perfusion will be quantified by fitting established biophysical models to arterial spin-labelling data, providing voxel-wise estimates of cerebral blood flow (CBF) in units of millilitres of blood per 100 g of tissue per minute.

##### White matter integrity and structural connectivity

White matter integrity will be assessed by fitting diffusion models to data from a diffusion-weighted sequence (60 directions *b* = 1000 s/mm^2^, and 60 directions *b* = 2200 s/mm^2^ interleaved with 13 T2-weighted *b* = 0 volumes, 2 × 2 × 2 mm image resolution, 8 min duration). Diffusion parameters of interest are fractional anisotropy and axial, radial, and mean diffusivity. Graph theoretical analysis will be performed using Brain Connectivity Toolbox to examine the integrity of white matter networks. Networks are a collection of nodes connected through pairwise relationships (edges). The topological features and the brain network’s behaviour can be characterised via a series of global and local network measures. The global network measures to be analysed will include (density, total weight), node-based (degree, clustering, path length), network organisation (scale-free, modular edge-based betweenness, multi-edge based (rich cub, motifs, path motifs), removal statistics (node, edge), and the whole system (Laplacian, eigenvector, centrality, modelling).

#### Additional brain MRI endpoints

Children who tolerate the core MRI protocol (awake) will be asked whether they are willing to undergo the following additional sequences: Multiparametric map sequence (MPM; 15 min) to assess tissue characteristics T2-Relaxation-Under-Spin-Tagging sequence (TRUST; 5 min) to assess oxygen-extraction fraction, and resting-state fMRI sequence (rsfMRI; 6 min) to assess functional connectivity (if children are awake).

#### Sleep

##### Overnight oximetry

When feasible, overnight oximetry will be collected at home to measure (i) mean and (ii) minimum oxygen saturation and (iii) the number of dips per hour.

#### Covariates

##### Demographic questionnaire

A caregiver will provide demographic information (e.g. age, sex, ethnicity, caregiver education, marital status).

##### Medical history

Study team members will obtain medical history (i.e. SCI) and laboratory results (i.e. haemoglobin) from local PIs taken as part of standard care from birth and 6 months after randomisation.

##### The Systemic Clinical Outcome and Routine Evaluation (SCORE-15) [92]

Caregivers will complete the SCORE-15, which is a 15-item measure assessing family processes and aspects of family functioning (e.g. ‘We are good at finding new ways to deal with things that are difficult’). Responses to items are rated on a 5-point Likert scale ranging from 1 = *describes my family very or extremely well*, to 5 = *describes my family—not at all*. The total score ranges from 15 to 75, with high scores indicating more family problems. Caregivers then rate the perceived burden of problems and family functioning on 3 10-point Likert scales. Scores are summed to obtain a level of family adjustment.

##### COVID-19 Exposure and Family Impact Survey (CEFIS) [93]

Because the SMILES trial will be conducted during the ongoing COVID-19 pandemic; caregivers will complete the CEFIS, which assesses aspects of the COVID-19 epidemic that are likely to impact families. The CEFIS contains subscales; part 1 (Exposure) consists of 25 items (yes/no responses), part 2 (Impact) consists of 12 items with 10 items using a 4-point scale rating impact on caregiver participant’s and family’s life and 2 items that use a 10-point distress scale. Part 3 is an open-ended question so that participants can expand upon their experiences. Higher scores denote more negative impact/exposure.

#### Plans to promote participant retention and complete follow-up {18b}

We will employ strategies to optimise recruitment and retention, including reminder phone calls, scheduling baseline, and follow-up visits convenient for caregivers, allowing caregivers to complete questionnaires online or by phone, and check-in with families during and after the trial.

#### Data management {19}

All documents will be stored safely at UCL GOS Institute of Child Health. For study-specific documents, other than the signed consent, the participant will be referred to by the study participant number/code, not by name. All data files will use a unique study assigned identifier codes. Electronic data files will be password protected, with access limited to study personnel. Informed consent documents will be maintained in locked storage cabinets within the PI’s locked office space at each site. Consent and permission forms will be kept separate from the participant’s data. Only the study staff will have access to the keys to the cabinets. Medical chart data will be collected by trained study staff under the supervision of the site PI. The full study database will be maintained on the UCL data haven web-based server.

Data quality will be maintained through double data entry from research team members and data quality checks that assess for conformance (does the data adhere to pre-specified values?), completeness (does missing data fit with expectations or our existing knowledge?), and plausibility (are the data values believable and within an expected range or distribution?) [94]. Data quality will be monitored by random inspection by the first author for the first 3–5 participants at each site and then quarterly. Inter-rater reliability among research team members will be computed for patient medical records and cognitive data. Any reliability issues will be addressed with additional training. Discrepancies will be resolved by checking source data and, if necessary, by returning to patient charts to correct any inaccuracies.

Source data and trial documentation will also be available to external auditors if and when required and in the event of a regulatory inspection. Access to the final data set will remain with the CI. Archiving will be authorised by the Sponsor following submission of the end of the study report. Essential documents will be retained for a minimum of 25 years after completion of the trial. These documents will be kept for longer if required by the applicable regulatory requirements.

#### Confidentiality {27}

Patient data will not be available to anyone not directly associated with the trial. All research team members have been trained in data safety and monitoring, privacy, and confidentiality, minimising risks related to privacy and confidentiality loss. The performance of research team members will be monitored to ensure the strictest standards. Patient identifiable data, including initials, date of birth, and NHS number, will be required for the registration process. The participants will be identified only by initials and a participant’s ID number on the case report form (CRF) and any electronic database. All documents will be stored securely and only accessed by trial staff and authorised personnel. The trial will comply with the Data Protection Act, which requires data to be anonymised as soon as it is practical. Data will be stored in a secure manner and in accordance with the Data Protection Act 2018.

#### Plans for collection, laboratory evaluation, and storage of biological specimens for genetic or molecular analysis in this trial/future use {33}

These are not applicable, no samples collected.

### Statistical methods

#### Statistical methods for primary and secondary outcomes {20a}

An excel database will take downloadable data from the tablet computer used for the cognitive evaluation. The pharmacist will maintain randomisation codes, and the research team will maintain basic demographics information. Analyses will be performed in the SPSS and R statistical packages [95]. Differences in our primary outcome of processing speed between treatment and control arms at 12 weeks will be assessed adjusting for the stratification factor (i.e. hydroxyurea) and baseline measures as appropriate. Secondary outcomes will be similarly compared using linear, logistic, or Poisson regression models applicable to the outcome type (numeric, binary, or count). The primary analyses will be intention-to-treat on those patients who consented to randomisation. We will use bias-corrected and accelerated 95% confidence intervals (CI) for all analyses as they adjust for possible bias and determine significance at an alpha level of *p* < .05 two-tailed.

#### Interim analyses {21b}

Interim analyses are not planned as unexpected serious adverse events related to the intervention are not expected. The trial participation for each patient is short, and they will be followed up in their local paediatric haematology centres.

#### Methods for additional analyses (e.g. subgroup analyses) {20b}

Analyses will consider how results differ according to age, whether the child had an SCI, oxygen content, overnight oxygen saturation, and sleep duration. Baseline demographics, age, sex, and most recent haemoglobin will be compared for those consenting to, and completing the trial and the non-consenters/non-completers to identify any potential biases and the generalisability of results.

Sensitivity analyses will also compare observations collected during higher UK COVID-19 restrictions versus lower/no COVID-19 restrictions. We will examine the impact of the COVID-19 pandemic on our trial outcomes by assessing the interaction between our treatment groups (montelukast versus placebo) and scores on the caregiver completed CEFIS in regression models. Finally, in addition to assessing hydroxyurea as a stratification factor, we will also determine if there is a hydroxyurea exposure × dose per kilogram effect on processing speed (e.g. does more prolonged hydroxyurea exposure after accounting for weight affect processing speed?)

#### Methods in analysis to handle protocol non-adherence and any statistical methods to handle missing data {20c}

All randomised participants will be included in analyses based on intention to treat. Missing data will be imputed under the assumption of the data being missing at random (MAR). Results will be compared with complete case analyses. The MAR assumption will be explored before the trial and before drop-outs occur. We will use multiple imputations for ITT analysis, but check the MAR assumption against reasons given for drop-out and complete case for per protocol to compare the results.

#### Plans to give access to the full protocol, participant-level data, and statistical code {31c}

The SMILES protocol is publicly available on clinicaltrials.gov. There are no current plans for granting public access to the participant-level dataset and statistical code.

### Oversight and monitoring

#### Composition of the coordinating centre and trial steering committee {5d}

The Trial Steering Committee responsibilities, in consultation with site PIs, include governance, protocol development and amendments, study design, planning, monitoring, and progress along with data collection and validation, and preparation of the manuscript for publication. No publications will be disseminated without the Trial Steering Committee’s prior approval. PIs at each participating site will be responsible for study conduct and day-to-day operations. The Trial Steering Committee consists of project coordinators, data managers, and research assistants (i.e. postdoctoral fellow, PhD and MSc students) and is led by the coordinating centre PI. Data analyses will be performed by the study statistician and a member of the Trial Steering Committee. A member of the Trial Steering Committee will meet bi-weekly with the participating site PIs. The Trial Steering Committee will meet once a month to discuss study progress.

#### Composition of the data monitoring committee, its role and reporting structure {21a}

This study has an external data monitoring committee (E-DMC). According to the DMC Charter, the DMC will be responsible for ongoing monitoring of participants’ efficacy and safety in the study. The recommendations made by the DMC to alter the conduct of the research will be forwarded to the Sponsor for a final decision. The Sponsor will forward such decisions to regulatory authorities, as appropriate. If the DMC recommendation is to halt the trial due to any other serious toxicity, then a substantial amendment must be submitted and approved by the MHRA before re-initiating recruitment. The DMC will be independent of the study team and will have no direct involvement in other trial aspects. The DMC will develop operation procedures in consultation with the sponsor, documented in the DMC charter.

#### Adverse event reporting and harms {22}

We will closely monitor participants for known potential and unexpected adverse events. The research coordinator will contact families during the first 1–2 weeks to determine any adverse events, especially behavioural, and then contact them again at 4 and 8 weeks by telephone. They will also be encouraged to contact the investigators or their local paediatricians/haematologists if they experience unexpected events. The number of children with treatment-related adverse events from montelukast will be measured by looking at all adverse events, including days of illness and days lost from preschool/school. Hospital admissions for pain, acute chest syndrome, stroke, planned surgery, splenectomy, and gallstones are expected in patients with SCA. These admissions will not be reported in this trial unless the CI believes that there is a causal relationship between the drug and the event. The management of any symptoms or exacerbations will be per usual clinical practice. The local PI or delegated research staff will discuss specific issues with families throughout the study. Any concerns which cannot be satisfied at a local level will be forwarded to the CI.

Adverse events will be recorded on case report forms by the study coordinator and encrypted and stored separately from other data collected. If the CI considers it necessary to know whether a participant is assigned to montelukast or placebo to manage a specific serious adverse event, the treatment may be unblinded for that participant. The GOSH pharmacy will access the randomisation schedule if required and supply the needed information to unblind the participant. The most frequently occurring adverse experiences consistent with the safety profile of montelukast include abdominal pain, somnolence, thirst, headache, vomiting, and psychomotor hyperactivity. If the participant is withdrawn due to an adverse event, we will arrange for follow-up visits or telephone calls until the adverse event has resolved or stabilised.

#### Frequency and plans for auditing trial conduct {23}

The Sponsor (UCL) is responsible for implementing quality control and quality assurance. A trial-specific monitoring plan will monitor the trial with the agreed plan. Initiation visits will be performed at each trial site before recruitment, followed by routine monitoring visits throughout the clinical trial. There will be study close-out visits once all data has been cleaned and queries have been resolved. We will submit protocol deviations that compromise safe drug administration or determine study endpoints to GOSH NHS Trust R&D Department. Protocol deviations will be included in the annual protocol review for the ethics committee. Source documents are original documents, data, and records from which participants’ CRF data are obtained. These include, but are not limited to, hospital records (from which medical history and previous and concurrent medication may be summarised into the CRF), clinical and office charts, laboratory and pharmacy records, microfiches, radiographs, and correspondence. Only members of the trial research team and the trial monitor will have direct access to the source data (i.e. hospital and pharmacy records) and trial documentation, which will be available to internal and external auditors if required. Access to the final data set will remain with the CI.

#### Plans for communicating important protocol amendments to relevant parties (e.g. trial participants, ethical committees) {25}

Any significant changes to the protocol outlined above (e.g. study objectives, study design, patient population, sample sizes, study procedures, or significant administrative aspects) will be written into a formal substantial amendment and approved by the ethics committee before implementation. New information that affects the risk/benefit assessment that results in changes to the consent form will require participants to be re-consented.

#### Dissemination plans {31a}

The study’s findings will be reported and disseminated at scientific conference presentations and in peer-reviewed journals and through engagement with medical providers, charities and community organisations, media, and the government. In particular, we will promote the outcomes of this study through lay-audience presentations, social media, and written feedback to patient support groups, as well as internal reports and articles on our lab webpages, which are publicly accessible.

## Discussion

Young children with SCA often have cognitive difficulties, specifically involving slowed processing speed [96–98]. These processing speed difficulties appear to be related to the brain’s deep white matter vulnerability to hypoxic-ischaemic injury [22]. Oxygen saturation often falls during sleep, especially if there is airway obstruction associated with the size of the adenoids in the nose as well as the tonsils, clinically detected as snoring [99]. There is preliminary evidence that suggests that children with SCA often have SDB associated with deficits in processing speed which early intervention could ameliorate [31]. However, the evidence base that widely used interventions, such as adenotonsillectomy and CPAP, to improve cognitive function is currently not strong, and consensus on treatment strategies has not yet been achieved [35, 38–40]. Montelukast is a safe, well-tolerated anti-inflammatory agent that mediates leukotrienes, which are key inflammatory mediators in the respiratory system. Several double-blind RCTs have demonstrated efficacy compared to placebo in the treatment of asthma in young children, and it reduces the size of the adenoids in children who do not have SCA [55–58]. However, we do not yet know whether montelukast would reduce adenoid size and improve processing speed in young children with SCA.

The SMILES trial aims to recruit 200 paediatric patients with SCA and SDB aged 3–7.99 years to assess the effect of montelukast on processing speed. Patients will be randomised to either montelukast or placebo for 12 weeks. Processing speed influences both intelligence and creativity, and these domains have an impact on school performance [100]. They often have reduced academic attainment compared to siblings and peers. Additional secondary outcomes will assess whether montelukast reduces adenoidal size and improves white matter integrity and executive function whilst seeing associated reduced respiratory and pain symptoms and improved sleep quality.

Potential challenges for the SMILES trial include recruiting a sufficient number of children into the study. The trial is being conducted within the context of the COVID-19 pandemic. Although vaccinations have begun, participants may be wary about taking part in research. As noted in our statistical analyses section, we can still answer important questions about adenoidal size, even with a reduced sample. Additionally, our research coordinator has kept in regular contact with many families, and they have indicated that if we begin the study, they would be willing to participate. Patients are also steadily referred to all collaborating centres. The research team has experience recruiting families (recently recruited 100 preschoolers); therefore, we feel the numbers proposed will still be achievable. In addition to recruitment challenges, caregivers may decline to complete all measures and tests. As such, we will prioritise our primary outcomes (i.e. processing speed) for patients and montelukast adherence monitoring for caregivers.

Hydroxyurea also improves oxygen saturation and is recommended for young children with SCA [101]. Families from the UK have expressed some concerns, so they may be reluctant to start this drug in asymptomatic young children. However, children with severe SCA may have been prescribed hydroxyurea, so we will account for this treatment in analyses. Cooperation in the MRI scanner may be an issue for younger children. The research team has experience applying neurophysiological methods with young children and will use these skills to adapt to an MRI ‘mock-scanner’ training regime. To maintain blinding, we will use a placebo and maintain the blind assessment of cognitive, oximetry, and imaging endpoints as we have done in previous studies of devices for SDB treatment.

Previous research indicates that interventions targeting hypoxic exposure may improve processing speed in young children with SCA [31, 44]. Thus, the SMILES trial will obtain data to determine if montelukast improves processing speed in young children with SCA and SDB. Findings from this study will also increase our understanding of whether montelukast is an effective treatment for young children with SCA. More broadly, data from this study may be utilised within the context of reducing emergency healthcare utilisation and improving quality of life for children with SCA.

## Trial status

SMILES protocol version 1.6, May 6, 2020. Recruitment will begin in April 2021 and is expected to be completed by September 2022.

## Abbreviations

BRIEF-2: Behavioral rating inventory of executive function
C-ACT: Childhood asthma control test
CBF: Cerebral blood flow
CI: Chief investigator
CEFIS: COVID-19 exposure and family impact survey
COVID-19: Coronavirus disease (SARS-CoV-2)
CPAP: Continuous positive airway pressure
CRF: Case report form
CSHQ: Children’s sleep habits questionnaire
DMC/DMSC: Data monitoring committee / data monitoring and safety committee
DTI: Diffusion tensor imaging
ESS: Epworth sleepiness scale
EudraCT: European clinical trials database
FLAIR: Fluid-attenuated inversion recovery
GOSH: Great Ormond street hospital
GSTH: Guys and St. Thomas hospital
NIMP: Impact project management
MAQ: Medical adherence questionnaire
MPM: Multiparametric map sequence
MRI: Magnetic resonance imaging
NHS: National health service
PedsQL: Pediatric quality of life
PSQ: Pediatric sleep questionnaire
PI: Principal investigator
R&D: NHS trust R&D department
RBC: Red blood cell
RCT: Randomised controlled trial
rsFMRI: Resting-state fMRI sequence
SCA: Sickle cell anaemia
SCD: Sickle cell disease
SCI: Silent cerebral infarct
SDB: Sleep-disordered breathing
SMILES: **S**tudy of montelukast in children with sickle cell disease
SO_2_: Oxygen saturation
SOP: Standard operating procedure
SCORE-15: Systemic clinical outcome and routine evaluation
TMF: Trial master file
TRUST: T2-relaxation-under-spin-tagging
UCL: University college London
WISC-V: Wechsler intelligence scale for children fifth edition
WPPSI-IV: Wechsler preschool & primary scale of intelligence fourth edition

## References

[1] Rees DC, Williams TN, Gladwin MT (2010). Sickle-cell disease. Lancet.

[2] Rackoff WR, Kunkel N, Silber JH, Asakura T, Ohene-Frempong K (1993). Pulse oximetry and factors associated with hemoglobin oxygen desaturation in children with sickle cell disease. Blood.

[3] Modell B, Darlison M (2008). Global epidemiology of haemoglobin disorders and derived service indicators. Bull World Health Organ.

[4] Platt OS, Brambilla DJ, Rosse WF, Milner PF, Castro O, Steinberg MH, Klug PP (1994). Mortality in sickle cell disease--life expectancy and risk factors for early death. N Engl J Med.

[5] Piel FB, Hay SI, Gupta S, Weatherall DJ, Williams TN (2013). Global burden of sickle cell anaemia in children under five, 2010–2050: modelling based on demographics, excess mortality, and interventions. PLoS Med.

[6] Telfer P, Coen P, Chakravorty S, Wilkey O, Evans J, Newell H, Smalling B, Amos R, Stephens A, Rogers D, Kirkham F (2007). Clinical outcomes in children with sickle cell disease living in England: a neonatal cohort in East London. Haematologica.

[7] DeBaun MR, Kirkham FJ (2016). Central nervous system complications and management in sickle cell disease: a review. Blood.

[8] DeBaun MR, Armstrong FD, McKinstry RC (2012). Silent cerebral infarcts: a review on a prevalent and progressive cause of neurologic injury in sickle cell anemia. Blood.

[9] Cancio MI, Helton KJ, Schreiber JE, Smeltzer MP, Kang G, Wang WC (2015). Silent cerebral infarcts in very young children with sickle cell anaemia are associated with a higher risk of stroke. Br J Haematol.

[10] Kawadler JM, Clayden JD, Clark CA, Kirkham FJ (2016). Intelligence quotient in paediatric sickle cell disease: a systematic review and meta-analysis. Dev Med Child Neurol.

[11] Prussien KV, Jordan LC, DeBaun MR (2019). Cognitive function in sickle cell disease across domains, cerebral infarct status, and the lifespan: A Meta-Analysis. J Pediatr Psychol.

[12] Hollocks MJ, Kok TB, Kirkham FJ, Gavlak J, Inusa BP, DeBaun MR, de Haan M (2012). Nocturnal oxygen desaturation and disordered sleep as a potential factor in executive dysfunction in sickle cell anemia. J Int Neuropsychol Soc.

[13] Noll RB, Stith L, Gartstein MA, Ris MD, Grueneich R, Vannatta K, Kalinyak K (2001). Neuropsychological functioning of youths with sickle cell disease: comparison with non-chronically ill peers. J Pediatr Psychol.

[14] Steen RG, Hu XJ, Elliott VE (2002). Kindergarten readiness skills in children with sickle cell disease: evidence of early neurocognitive damage?. J Child Neurol.

[15] Tarazi RA, Grant ML, Ely E, Barakat LP (2007). Neuropsychological functioning in preschool-age children with sickle cell disease: the role of illness-related and psychosocial factors. Child Neuropsychol.

[16] Thompson RJ, Armstrong FD, Link CL (2003). A prospective study of the relationship over time of behavior problems, intellectual functioning, and family functioning in children with sickle cell disease: a report from the Cooperative Study of Sickle Cell Disease. J Pediatr Psychol.

[17] Hogan AM, Telfer PT, Kirkham FJ, Haan M (2013). Precursors of executive function in infants with sickle cell anemia. J Child Neurol.

[18] Schatz J, Finke RL, Kellett JM, Kramer JH (2002). Cognitive functioning in children with sickle cell disease: a meta-analysis. J Pediatr Psychol.

[19] Wang W, Enos L, Gallagher D, Thompson R, Guarini L, Vichinsky E, Wright E, Zimmerman R, Armstrong FD, Cooperative Study of Sickle Cell Disease (2001). Neuropsychologic performance in school-aged children with sickle cell disease: a report from the Cooperative Study of Sickle Cell Disease. J Pediatr.

[20] Hood AM, King AA, Fields ME, Ford AL, Guilliams KP, Hulbert ML, Lee JM, White DA (2019). Higher executive abilities following a blood transfusion in children and young adults with sickle cell disease. Pediatr Blood Cancer.

[21] Hardy SJ, Hardy KK, Schatz JC, Thompson AL, Meier ER (2016). Feasibility of home-based computerized working memory training with children and adolescents with sickle cell disease. Pediatr Blood Cancer.

[22] Stotesbury H, Kirkham FJ, Kölbel M, Balfour P, Clayden JD, Sahota S, Sakaria S, Saunders DE, Howard J, Kesse-Adu R, Inusa B, Pelidis M, Chakravorty S, Rees DC, Awogbade M, Wilkey O, Layton M, Clark CA, Kawadler JM (2018). White matter integrity and processing speed in sickle cell anemia. Neurology.

[23] Daly B, Kral MC, Brown RT, Elkin D, Madan-Swain A, Mitchell M, Crosby L, DeMatteo D, LaRosa A, Jackson S (2012). Ameliorating attention problems in children with sickle cell disease: a pilot study of methylphenidate. J Dev Behav Pediatr.

[24] Hood AM, Reife I, King AA, White DA (2019). Brief screening measures identify risk for psychological difficulties among children with sickle cell disease. J Clin Psychol Med Settings.

[25] Kirkham FJ, Hewes DKM, Prengler M, Wade A, Lane R, Evans JPM (2001). Nocturnal hypoxaemia and central-nervous-system events in sickle-cell disease. Lancet.

[26] Makani J, Kirkham FJ, Komba A, Ajala-Agbo T, Otieno G, Fegan G, Williams TN, Marsh K, Newton CR (2009). Risk factors for high cerebral blood flow velocity and death in Kenyan children with sickle cell anaemia: role of haemoglobin oxygen saturation and febrile illness. Br J Haematol.

[27] Sommet J, Alberti C, Couque N, Verlhac S, Haouari Z, Mohamed D, François M, Missud F, Holvoet L, Elmaleh M, Ithier G, Denjean A, Elion J, Baruchel A, Benkerrou M (2016). Clinical and haematological risk factors for cerebral macrovasculopathy in a sickle cell disease newborn cohort: a prospective study. Br J Haematol.

[28] Kawadler JM, Kirkham FJ, Clayden JD, Hollocks MJ, Seymour EL, Edey R, Telfer P, Robins A, Wilkey O, Barker S, Cox TCS, Clark CA (2015). White matter damage relates to oxygen saturation in children with sickle cell anemia without silent cerebral infarcts. Stroke.

[29] King AA, Strouse JJ, Rodeghier MJ, Compas BE, Casella JF, McKinstry RC, Noetzel MJ, Quinn CT, Ichord R, Dowling MM, Miller JP, DeBaun MR (2014). Parent education and biologic factors influence on cognition in sickle cell anemia. Am J Hematol.

[30] Hogan AM, Pitten Cate IM, Vargha-Khadem F (2006). Physiological correlates of intellectual function in children with sickle cell disease: hypoxaemia, hyperaemia and brain infarction. Dev Sci.

[31] Marshall MJ, Bucks RS, Hogan AM, Hambleton IR, Height SE, Dick MC, Kirkham FJ, Rees DC (2009). Auto-adjusting positive airway pressure in children with sickle cell anemia: results of a phase I randomized controlled trial. Haematologica.

[32] De A, Manwani D, Rastogi D (2018). Airway inflammation in sickle cell disease—a translational perspective. Pediatric Pulmonology.

[33] Katz ES, D’Ambrosio CM (2008). Pathophysiology of pediatric obstructive sleep apnea. Proc Am Thoracic Soc.

[34] Hamada M, Iida M, Nota J, Matsumoto N, Sawada S, Mukushita N, Washizu Y, Shimasaki M, Doi T (2015). Safety and efficacy of adenotonsillectomy for obstructive sleep apnea in infants, toddlers and preschool children. Auris Nasus Larynx.

[35] Marcus CL, Moore RH, Rosen CL, Giordani B, Garetz SL, Taylor HG, Mitchell RB, Amin R, Katz ES, Arens R, Paruthi S, Muzumdar H, Gozal D, Thomas NH, Ware J, Beebe D, Snyder K, Elden L, Sprecher RC, Willging P, Jones D, Bent JP, Hoban T, Chervin RD, Ellenberg SS, Redline S, Childhood Adenotonsillectomy Trial (CHAT) (2013). A randomized trial of adenotonsillectomy for childhood sleep apnea. N Engl J Med.

[36] Marcus CL, Radcliffe J, Konstantinopoulou S, Beck SE, Cornaglia MA, Traylor J, DiFeo N, Karamessinis LR, Gallagher PR, Meltzer LJ (2012). Effects of positive airway pressure therapy on neurobehavioral outcomes in children with obstructive sleep apnea. Am J Respir Crit Care Med.

[37] Meltzer EO, Philip G, Weinstein SF, LaForce CF, Malice MP, Dass SB, Santanello NC, Reiss TF (2005). Montelukast effectively treats the nighttime impact of seasonal allergic rhinitis. Am J Rhinol.

[38] Venekamp RP, Hearne BJ, Chandrasekharan D, Blackshaw H, Lim J, Schilder AGM, et al. Tonsillectomy or adenotonsillectomy versus non-surgical management for obstructive sleep-disordered breathing in children. Cochrane Database of Systematic Reviews. 2015:1465–858. 10.1002/14651858.CD011165.pub2. 10.1002/14651858.CD011165.pub2 PMC9242010 26465274

[39] Biggs SN, Vlahandonis A, Anderson V, Bourke R, Nixon GM, Davey MJ, Horne RSC (2014). Long-term changes in neurocognition and behavior following treatment of sleep disordered breathing in school-aged children. Sleep.

[40] Landau YE, Bar-Yishay O, Greenberg-Dotan S, Goldbart AD, Tarasiuk A, Tal A (2012). Impaired behavioral and neurocognitive function in preschool children with obstructive sleep apnea. Pediatr Pulmonol.

[41] Rosen CL, Debaun MR, Strunk RC, Redline S, Seicean S, Craven DI, Gavlak JCD, Wilkey O, Inusa B, Roberts I, Goodpaster RL, Malow B, Rodeghier M, Kirkham FJ (2014). Obstructive sleep apnea and sickle cell anemia. Pediatrics.

[42] Kaleyias J, Mostofi N, Grant M, Coleman C, Luck L, Dampier C, Kothare SV (2008). Severity of obstructive sleep apnea in children with sickle cell disease. J Pediatr Hematol Oncol.

[43] Mascarenhas MI, Loureiro HC, Ferreira T, Dias A (2015). Sleep pathology characterization in sickle cell disease: Case-control study. Pediatr Pulmonol.

[44] Machaalani R, Evans CA, Waters KA (2016). Objective adherence to positive airway pressure therapy in an Australian paediatric cohort. Sleep Breath.

[45] Inusa B, Stotesbury H, Koelbel M (2018). Prevention of morbidity in Sickle Cell Disease Phase II (POMS 2b paediatric cohort): improvement of pain and quality of life in children with Sickle Cell Disease with auto-adjusting Continuous Positive Airways Pressure. Br J Haematol.

[46] Slee A, Kawadler J, Koelbel M (2017). Prevention of morbidity in Sickle Cell Disease Phase II (Improvement of Cognition in children with Sickle Cell Disease with Auto-adjusting Continuous Positive Airways Pressure: Phase II) (POMS 2b paediatric cohort). Blood.

[47] Sawyer AM, Gooneratne N, Marcus CL (2011). A systematic review of CPAP adherence across age groups: clinical and empiric insights for developing CPAP adherence interventions. Sleep Med Rev.

[48] Knight-Perry J, Debaun MR, Strunk RC (2009). Leukotriene pathway in sickle cell disease: a potential target for directed therapy. Expert Review of Hematology.

[49] Field JJ, Strunk RC, Knight-Perry JE, Blinder MA, Townsend RR, DeBaun MR (2009). Urinary cysteinyl leukotriene E4 significantly increases during pain in children and adults with sickle cell disease. Am J Hematol.

[50] Paggiaro P, Bacci E (2011). Montelukast in asthma: a review of its efficacy and place in therapy. Ther Adv Chronic Dis.

[51] Leff JA, Busse WW, Pearlman D, Bronsky EA, Kemp J, Hendeles L, Dockhorn R, Kundu S, Zhang J, Seidenberg BC, Reiss TF (1998). Montelukast, a leukotriene-receptor antagonist, for the treatment of mild asthma and exercise-induced bronchoconstriction. N Engl J Med.

[52] Kemp JP, Dockhorn RJ, Shapiro GG, Nguyen HH, Reiss TF, Seidenberg BC, Knorr B (1998). Montelukast once daily inhibits exercise-induced bronchoconstriction in 6- to 14-year-old children with asthma. J Pediatr.

[53] Okubo K, Inoue Y, Numaguchi H, Tanaka K, Saito I, Oshima N, Matsumoto Y, Prohn M, Mehta A, Nishida C, Philip G (2016). Montelukast in the treatment of perennial allergic rhinitis in paediatric Japanese patients; an open-label clinical trial. J Drug Assess.

[54] Skoner D (2001). Montelukast in 2- to 5-year-old children with asthma. Pediatr Pulmonol.

[55] Bérubé D, Djandji M, Sampalis JS (2014). Effectiveness of montelukast administered as monotherapy or in combination with inhaled corticosteroid in pediatric patients with uncontrolled asthma: a prospective cohort study. Allergy Asthma Clin Immunol.

[56] Becker A (2000). Clinical evidence with montelukast in the management of chronic childhood asthma. Drugs.

[57] Goldbart AD, Greenberg-Dotan S, Tal A (2012). Montelukast for children with obstructive sleep apnea: a double-blind, placebo-controlled study. Pediatrics.

[58] Knorr B, Franchi LM, Bisgaard H, Vermeulen JH, LeSouef P, Santanello N, Michele TM, Reiss TF, Nguyen HH, Bratton DL (2001). Montelukast, a leukotriene receptor antagonist, for the treatment of persistent asthma in children aged 2 to 5 years. Pediatrics.

[59] Bisgaard H, Zielen S, Garcia-Garcia ML, Johnston SL, Gilles L, Menten J, Tozzi CA, Polos P (2005). Montelukast reduces asthma exacerbations in 2- to 5-year-old children with intermittent asthma. Am J Respir Crit Care Med.

[60] Jackman AR, Biggs SN, Walter LM, Embuldeniya US, Davey MJ, Nixon GM, Anderson V, Trinder J, Horne RSC (2012). Sleep-disordered breathing in preschool children is associated with behavioral, but not cognitive, impairments. Sleep Med.

[61] Steinberg MH (2005). Predicting clinical severity in sickle cell anaemia. Br J Haematol.

[62] Singh P, Sharma B (2016). Reversal in cognition impairments, cholinergic dysfunction, and cerebral oxidative stress through the modulation of ryanodine receptors (RyRs) and cysteinyl leukotriene-1 (CysLT1) receptors. Curr Neurovasc Res.

[63] Marschallinger J, Schäffner I, Klein B, Gelfert R, Rivera FJ, Illes S, Grassner L, Janssen M, Rotheneichner P, Schmuckermair C, Coras R, Boccazzi M, Chishty M, Lagler FB, Renic M, Bauer HC, Singewald N, Blümcke I, Bogdahn U, Couillard-Despres S, Lie DC, Abbracchio MP, Aigner L (2015). Structural and functional rejuvenation of the aged brain by an approved anti-asthmatic drug. Nat Commun.

[64] Fumagalli M, Lecca D, Abbracchio MP (2016). CNS remyelination as a novel reparative approach to neurodegenerative diseases: the roles of purinergic signaling and the P2Y-like receptor GPR17. Neuropharmacology.

[65] Wang L, Du C, Lv J (2011). Antiasthmatic drugs targeting the cysteinyl leukotriene receptor 1 alleviate central nervous system inflammatory cell infiltration and pathogenesis of experimental autoimmune encephalomyelitis. J Immunol.

[66] Tamada T, Ichinose M, Page C, Barnes P (2016). Leukotriene receptor antagonists and antiallergy drugs. Handbook of Experimental Pharmacology.

[67] Bacharier LB, Boner A, Carlsen KCL, et al. Diagnosis and treatment of asthma in childhood: a PRACTALL consensus report. In: Allergy: European Journal of Allergy and Clinical Immunology. 2008, pp. 5–34. 10.1111/j.1398-9995.2007.01586.x 18053013

[68] Kheirandish-Gozal L, Bandla HPR, Gozal D (2016). Montelukast for children with obstructive sleep apnea: results of a double-blind, randomized, placebo-controlled trial. Ann Am Thorac Soc.

[69] Shokouhi F, Meymaneh Jahromi A, Majidi MR, Salehi M (2015). Montelukast in adenoid hypertrophy: its effect on size and symptoms. Iran J Otorhinolaryngol.

[70] Wang WC, Ware RE, Miller ST, Iyer RV, Casella JF, Minniti CP, Rana S, Thornburg CD, Rogers ZR, Kalpatthi RV, Barredo JC, Brown RC, Sarnaik SA, Howard TH, Wynn LW, Kutlar A, Armstrong FD, Files BA, Goldsmith JC, Waclawiw MA, Huang X, Thompson BW, BABY HUG investigators (2011). A multicenter randomised controlled trial of hydroxyurea (hydroxycarbamide) in very young children with sickle cell anaemia. Lancet.

[71] Charache S, Terrin ML, Moore RD, Dover GJ, Barton FB, Eckert SV, McMahon RP, Bonds DR (1995). Effect of hydroxyurea on the frequency of painful crises in sickle cell anemia. N Engl J Med.

[72] Paediatric Formulary Committee. BNF for Children (BNFC) 2019-2020. Pharmaceutical Press, https://vnras.com/wp-content/uploads/2020/03/BNF-for-Children-BNFC-2019-2020-1.pdf (2019).

[73] Weintraub S, Dikmen SS, Heaton RK, Tulsky DS, Zelazo PD, Bauer PJ, Carlozzi NE, Slotkin J, Blitz D, Wallner-Allen K, Fox NA, Beaumont JL, Mungas D, Nowinski CJ, Richler J, Deocampo JA, Anderson JE, Manly JJ, Borosh B, Havlik R, Conway K, Edwards E, Freund L, King JW, Moy C, Witt E, Gershon RC (2013). Cognition assessment using the NIH Toolbox. Neurology.

[74] Hood AM, Reife I, King AA, White DA (2020). Brief screening measures identify risk for psychological difficulties among children with sickle cell disease. J Clin Psychol Med Settings.

[75] Ludwig NN, Sil S, Khowaja MK, Cohen LL, Dampier C (2018). Executive functioning mediates the relationship between pain coping and quality of life in youth with sickle cell disease. J Pediatr Psychol.

[76] Schlenz AM, Schatz J, Roberts CW (2016). Examining biopsychosocial factors in relation to multiple pain features in pediatric sickle cell disease. J Pediatr Psychol.

[77] Hogan AM, Hill CM, Harrison D, et al. Cerebral blood flow velocity and cognition in children before and after adenotonsillectomy. Pediatrics; 122. 10.1542/peds.2007-2540 18595989

[78] Slee A, Stotesbury H, Kawadler J (2019). Prevention of morbidity in Sickle Cell Disease Phase 2 (POMS 2b pediatric): improvement of cognition in children with sickle cell disease with auto-adjusting continuous positive airways pressure: a single-blind, randomized, controlled phase II trial. Br J Haematol.

[79] DeBaun MR, Gordon M, McKinstry RC (2014). Controlled trial of transfusions for silent cerebral infarcts in sickle cell anemia. N Engl J Med.

[80] Goldbart AD, Goldman JL, Li RC, Brittian KR, Tauman R, Gozal D (2004). Differential expression of cysteinyl leukotriene receptors 1 and 2 in tonsils of children with obstructive sleep apnea syndrome or recurrent infection. Chest.

[81] Wahlstrom D, Raiford SE, Breaux KC, et al. Contemporary intellectual assessment: theories, tests, and issues. In: Flanagan D, EM MD, editors. The Wechsler Preschool and Primary Scale of Intelligence—Fourth Edition, Wechsler Intelligence Scale for Children—Fifth Edition, and Wechsler Individual Achievement Test—Third Edition. New York: The Guilford Press; 2018. p. 245–82.

[82] Flanagan DP, Alfonso VC. Essentials of WISC-V assessment. 1st ed. Hoboken: Wiley; 2017.

[83] Wahlstrom DA, Daniel PM, Weiss LG. Digital assessment with Q-interactive. WISC-V Clin Use Interpret. 2019;417. 10.1016/B978-0-12-815744-2.00012-4.

[84] Gioia GA, Isquith PK, Guy SC, et al. BRIEF-2: Behavior Rating Inventory of Executive Function. Psychological Assessment Resources, 2015.

[85] Panepinto JA, Torres S, Bendo CB, McCavit TL, Dinu B, Sherman-Bien S, Bemrich-Stolz C, Varni JW (2013). PedsQL^TM^ sickle cell disease module: feasibility, reliability, and validity. Pediatr Blood Cancer.

[86] Beverung LM, Varni JW, Panepinto JA (2015). Clinically meaningful interpretation of pediatric health-related quality of life in sickle cell disease. J Pediatr Hematol Oncol.

[87] Owens JA, Spirito A, McGuinn M (2000). The Children’s Sleep Habits Questionnaire (CSHQ): psychometric properties of a survey instrument for school-aged children. Sleep-New York.

[88] Chervin RD, Hedger K, Dillon JE, Pituch KJ (2000). Pediatric sleep questionnaire (PSQ): validity and reliability of scales for sleep-disordered breathing, snoring, sleepiness, and behavioral problems. Sleep Med.

[89] Johns MW (1991). A new method for measuring daytime sleepiness: the Epworth sleepiness scale. Sleep.

[90] Liu AH, Zeiger R, Sorkness C, Mahr T, Ostrom N, Burgess S, Rosenzweig JC, Manjunath R (2007). Development and cross-sectional validation of the Childhood Asthma Control Test. J Allergy Clin Immunol.

[91] Knobel H, Alonso J, Casado JL, Collazos J, González J, Ruiz I, Kindelan JM, Carmona A, Juega J, Ocampo A, GEEMA Study Group (2002). Validation of a simplified medication adherence questionnaire in a large cohort of HIV-infected patients: the GEEMA Study. Aids.

[92] Stratton P, Bland J, Janes E, Lask J (2010). Developing an indicator of family function and a practicable outcome measure for systemic family and couple therapy: the SCORE. J Fam Ther.

[93] Kazak AE, Alderfer M, Enlow PT, et al. COVID-19 exposure and family impact scales: factor structure and initial psychometrics. J Pediatr Psychol. Epub ahead of print 22 March 2021. DOI: 10.1093/jpepsy/jsab026. 10.1093/jpepsy/jsab026 PMC8083683 33749794

[94] Lee K, Weiskopf N, Pathak J. A framework for data quality assessment in clinical research datasets. In: AMIA Annual Symposium Proceedings: American Medical Informatics Association; 2017. p. 1080. Accessed 21 Mar 2019. PMC5977591 29854176

[95] R Core Team. R: A Language and Environment for Statistical Computing, http://www.r-project.org (2018).

[96] Crawford RD, Jonassaint CR (2016). Adults with sickle cell disease may perform cognitive tests as well as controls when processing speed is taken into account: a preliminary case–control study. J Adv Nurs.

[97] Land V, Hijmans CT, Ruiter M (2015). Volume of white matter hyperintensities is an independent predictor of intelligence quotient and processing speed in children with sickle cell disease. Br J Haematol.

[98] Bockenmeyer J, Chamboredon E, Missud F, Benkerrou M, Holvoët L, Ithier G, Lescoeur B, Yakouben K, Ouachée-Chardin M, Rohrlich PS, Duval M, Baruchel A, Dalle JH (2013). Development of psychological and intellectual performance in transplanted sickle cell disease patients: a prospective study from pretransplant period to 5 years after HSCT. Arch Pediatr.

[99] Greenfeld M, Tauman R, DeRowe A, Sivan Y (2003). Obstructive sleep apnea syndrome due to adenotonsillar hypertrophy in infants. Int J Pediatr Otorhinolaryngol.

[100] Rindermann H, Neubauer AC (2004). Processing speed, intelligence, creativity, and school performance: testing of causal hypotheses using structural equation models. Intelligence.

[101] Wang WC, Ware RE, Miller ST, Iyer RV, Casella JF, Minniti CP, Rana S, Thornburg CD, Rogers ZR, Kalpatthi RV, Barredo JC, Brown RC, Sarnaik SA, Howard TH, Wynn LW, Kutlar A, Armstrong FD, Files BA, Goldsmith JC, Waclawiw MA, Huang X, Thompson BW, BABY HUG investigators (2011). Hydroxycarbamide in very young children with sickle-cell anaemia: a multicentre, randomised, controlled trial (BABY HUG). Lancet.

